# Supplementary material for: Plasma Virome Reveals Blooms and Transmission of Anellovirus in Intravenous Drug Users with HIV-1, HCV, and/or HBV Infections
Source: Microbiol Spectr. 2022 Jun 27;10(4):e01447-22. doi: 10.1128/spectrum.01447-22 (PMC9431549; doi:10.1128/spectrum.01447-22)
Supplement: Supplemental file 1 — Supplemental material. Download spectrum.01447-22-s0001.pdf, PDF file, 1.2 MB [file spectrum.01447-22-s0001.pdf]

Supplementary Tables

Table S1. Comparison between metagenomic sequencing and qPCR results.

PCR positive	NGS positive	Recovering rate by NGS
HIV-1 (n=26)	24	92.3%
HCV (n=11)	11	100%
HIV-1&HCV (n=29)	28	96.5%
HBV (n=10)	9	90%

Table S2. Anellovirus sequences from those clusters that each sequence shared identity over 95%.

Names	
Torque teno virus 10	*alpha*
TTV-like mini virus	*beta*
Torque teno midi virus 5	*gamma*
Torque teno midi virus 7	*gamma*
Torque teno midi virus 14	*gamma*
Torque teno virus	unclassified
Anelloviridae sp.	unclassified
Opossum torque teno virus	unclassified

Table S3. Relatedness of Anelloviridae between individuals within or out of transmission clusters (TC). Anellovirus reads that could be mapped to corresponding anellovirus contigs using stringent threshold were calculated, and data were shown as the proportion of recovered anellovirus reads within/out of TC relative to cognate anellovirus reads (anellovirus reads that mapped to contigs from the same individual). Relatedness of anellovirus reads within TC versus unrelated individuals was compared with Wilcoxon matched-pairs signed rank test.

	Sample ID	Percentage of shared anellovirus numbers	Cognate anellovirus reads	Within TC	Out of TC (Unrelated individual)	Within TC versus Unrelated (*p* value)
TC1	E11	E11/D15, 69%	209214	0.456	0.144	*p*=0.0005
	D15		496973	0.725	0.405	
TC2	F20	F20/G2, 79%	69757	0.276	0.127	
	G2	F20/G4, 64%	50314	0.517	0.464	
	G4	G2/G4, 57%	82248	0.246	0.210	
TC3	G9	G9/F9, 100%	43754	0.762	0.390	
	F9	G9/F10,100%	374248	0.623	0.294	
	F10	F9/F10, 98%	1472106	0.508	0.311	
TC4	G15	G15/G3, 94%	122943	0.416	0.261	
	G3		475060	0.409	0.340	
TC5	D12	D12/D13, 87%	761099	0.646	0.485	
	D13		439474	0.662	0.344	

Table S4. The detection of several neglected or emerging viruses in IDUs. Reads number of human pegivirus H (HPgV-2), hepatitis delta virus (HDV) and a novel circovirus are shown in each individual.

	#1	#2	#3	#4	#5	#6
HPgV-C	47149	207076			50	
HIV-1	14	264	98	5401	227	357
HCV	340	104	258	3157	3	20
HDV	7227	2069				
HPgV-2			54	470	12	
Novel Circovirus						718

Supplementary Figures

Figure S1. Comparison between metagenomic sequencing and qPCR. Spearman's correlation for HIV-1, HCV, HBV (a) and *anelloviridae* (b) was calculated to determine the correlation between the qPCR (Ct values) and metagenomic sequencing results (RPM).

Figure S2. Relative abundance of main vertebrate viruses (a) and prokaryotic viruses (bacteriophages)(b) by individual.

Figure S3. Influence of age (top) and duration of drug use (bottom) on the blood viral composition. Spearman's correlation was analyzed between age/duration of drug use and viral reads (RPM), Richness, Shannon index.

Figure S4. Number of anelloviruses detected in each individual (a), each bar represents the percentage of samples positive for given numbers of anelloviruses. Coexistence of anelloviruses in healthy controls and IDUs (b), each color represents the percentage of samples positive for given numbers of anelloviruses, number of samples were shown on each bar/color stack. Number of samples positive for anelloviruses that detected in HIV-1/HCV/HBV positive IDUs from Fig. 5c; orange, blue and red colors indicate alpha, beta, gamma genus of anellovirus, grey indicates unclassified anellovirus (c). Co-occurrence network of anelloviruses that detected in HIV-1/HCV/HBV positive IDUs are highlighted (d). TTCV, torque teno chlorocebus virus.

Figure S5. Number of positive samples positive for pegivirus, most pegiviruses were detected in IDUs with major viral infections (a). Correlations of the abundance of pegivirus with HIV-1(b), HCV(c) and anellovirus (d).

Figure S6. Relative abundance (calculated by RPM) of each anellovirus within each transmission cluster (TC) in Figure 6.

Figure S1

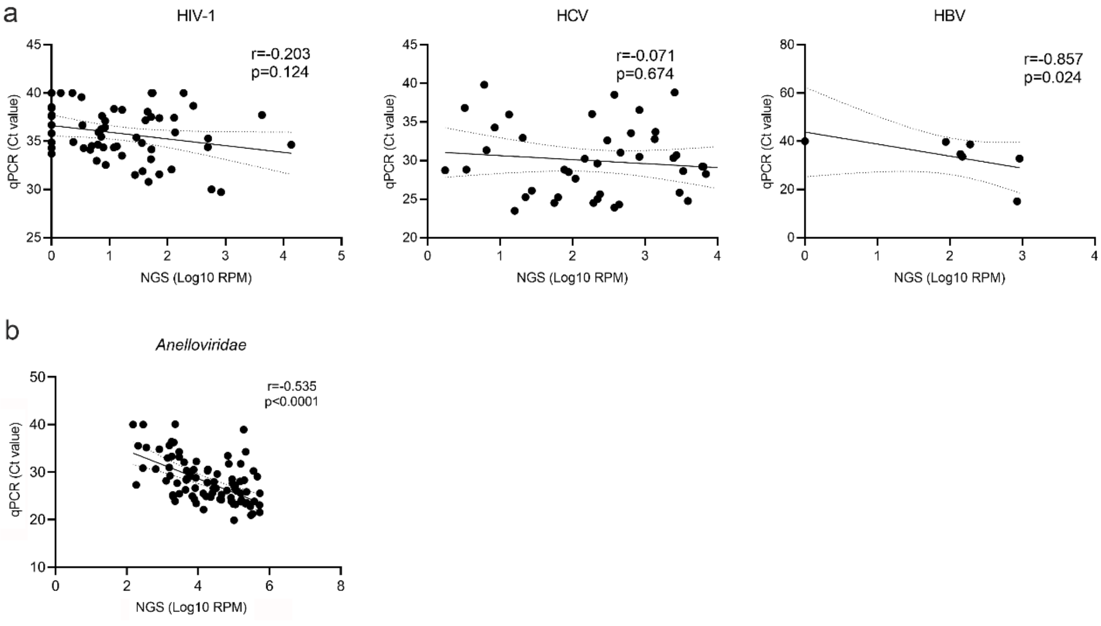
a
HIV-1
r=-0.203
p=0.124
qPCR (Ct value)
NGS (Log10 RPM)
HCV
r=-0.071
p=0.674
qPCR (Ct value)
NGS (Log10 RPM)
HBV
r=-0.857
p=0.024
qPCR (Ct value)
NGS (Log10 RPM)
b
Anelloviridae
r=-0.535
p<0.0001
qPCR (Ct value)
NGS (Log10 RPM)

Figure S2

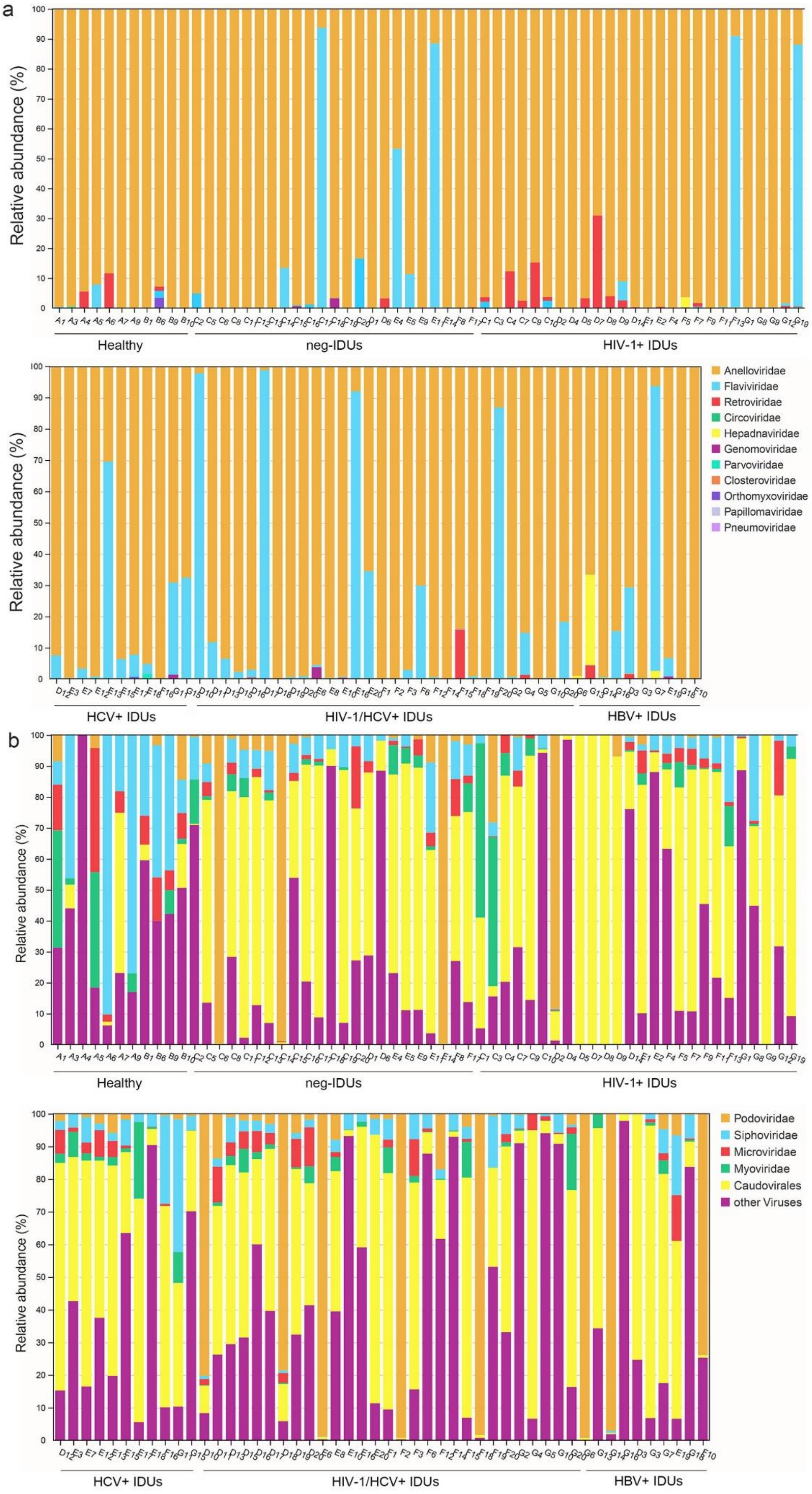
a
Relative abundance (%)
Healthy
neg-IDUs
HIV-1+ IDUs
HCV+ IDUs
HIV-1/HCV+ IDUs
HBV+ IDUs
Anelloviridae
Flaviviridae
Retroviridae
Circoviridae
Hepadnaviridae
Genomoviridae
Parvoviridae
Closteroviridae
Orthomyxoviridae
Papillomaviridae
Pneumoviridae
b
Podoviridae
Siphoviridae
Microviridae
Myoviridae
Caudovirales
other Viruses

Figure S3

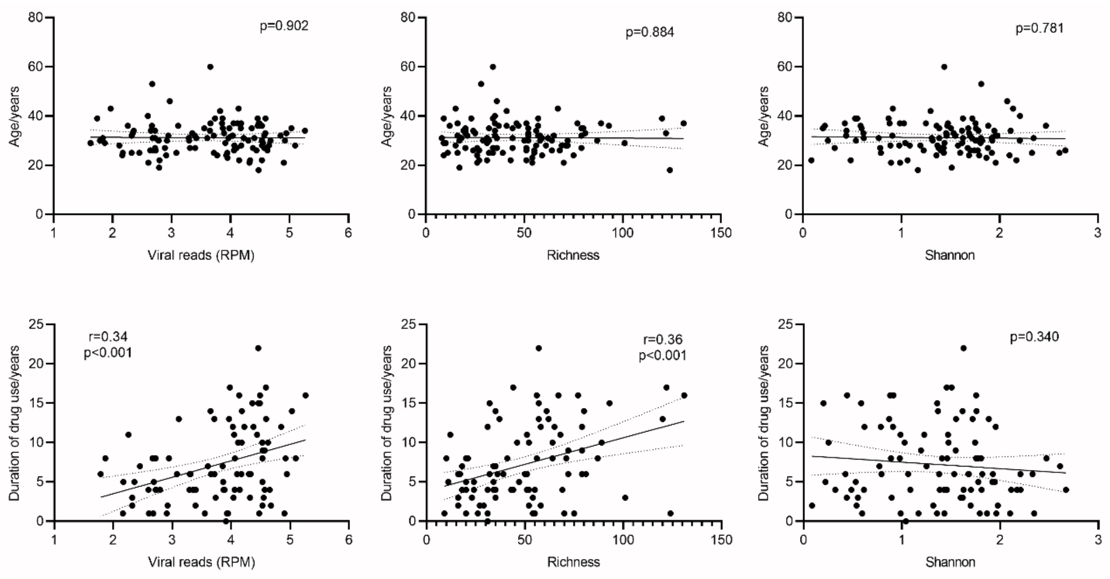

p=0.902
Age/years
Viral reads (RPM)
p=0.884
Age/years
Richness
p=0.781
Age/years
Shannon
r=0.34
p<0.001
Duration of drug use/years
Viral reads (RPM)
r=0.36
p<0.001
Duration of drug use/years
Richness
p=0.340
Duration of drug use/years
Shannon

Figure S4

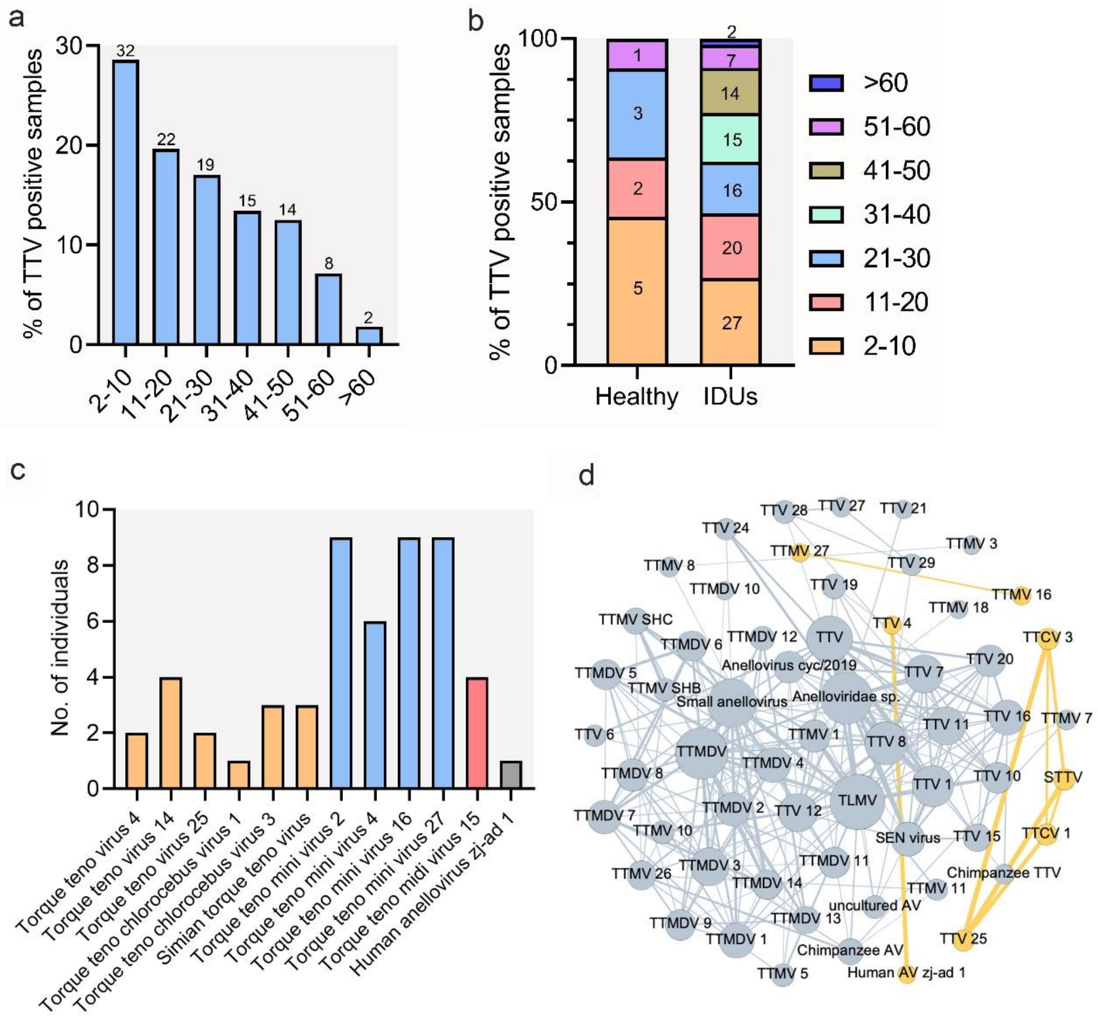
a
% of TTV positive samples
0
10
20
30
32
22
19
15
14
8
2
2-10
11-20
21-30
31-40
41-50
51-60
>60
b
% of TTV positive samples
0
50
100
1
3
2
5
2
7
14
15
16
20
27
Healthy
IDUs
>60
51-60
41-50
31-40
21-30
11-20
2-10
c
No. of individuals
0
2
4
6
8
10
Torque teno virus 4
Torque teno virus 14
Torque teno virus 25
Torque teno chlorocebus virus 1
Torque teno chlorocebus virus 3
Simian torque teno virus
Torque teno mini virus 2
Torque teno mini virus 4
Torque teno mini virus 16
Torque teno mini virus 27
Torque teno midi virus 15
Human anellovirus zj-ad 1
d
TTV 28
TTV 27
TTV 21
TTV 24
TTMV 27
TTMV 3
TTMV 8
TTV 29
TTMDV 10
TTV 19
TTMV 16
TTMV 18
TTMV SHC
TTMDV 12
TTV
TTV 4
TTCV 3
TTMDV 6
Anellovirus cyc/2019
TTV 20
TTMDV 5
TTV 7
TTMV SHB
Small anellovirus
Anelloviridae sp.
TTV 16
TTMV 7
TTV 11
TTV 6
TTMV 1
TTV 8
TTMDV
TTMDV 4
TTMDV 8
TTV 10
STTV
TTV 1
TLMV
TTMDV 2
TTV 12
TTMDV 7
TTMV 10
SEN virus
TTV 15
TTCV 1
TTMDV 3
TTMDV 11
TTMV 26
Chimpanzee TTV
TTMDV 14
TTMV 11
uncultured AV
TTMDV 13
TTMDV 9
TTV 25
TTMDV 1
Chimpanzee AV
TTMV 5
Human AV zj-ad 1

Figure S5

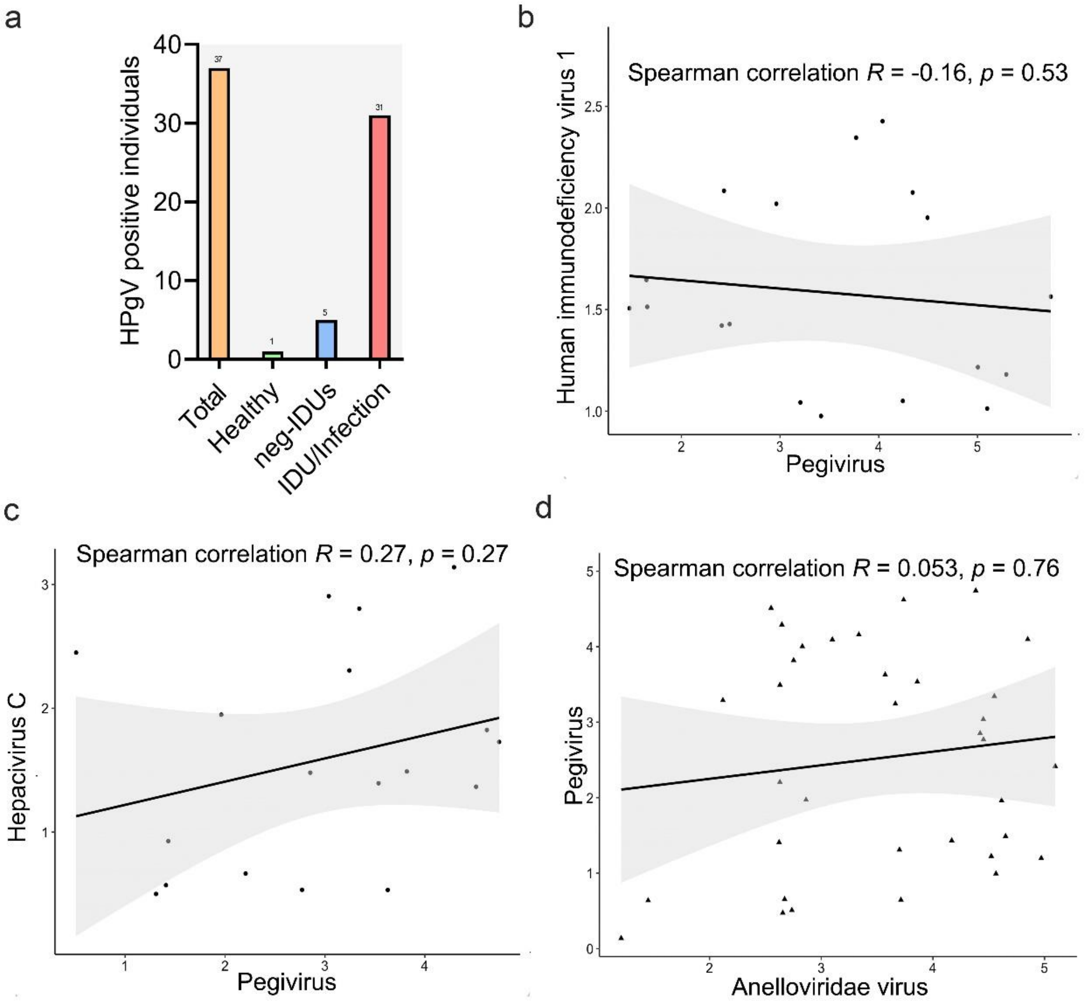
a
b
c
d
HPgV positive individuals
40
30
20
10
0
37
1
5
31
Total
Healthy
neg-IDUs
IDU/Infection
Spearman correlation R = -0.16, p = 0.53
Human immunodeficiency virus 1
2.5
2.0
1.5
1.0
2
3
4
5
Pegivirus
Spearman correlation R = 0.27, p = 0.27
Hepacivirus C
3
2
1
1
2
3
4
Pegivirus
Spearman correlation R = 0.053, p = 0.76
Pegivirus
5
4
3
2
1
0
2
3
4
5
Anelloviridae virus

Figure S6

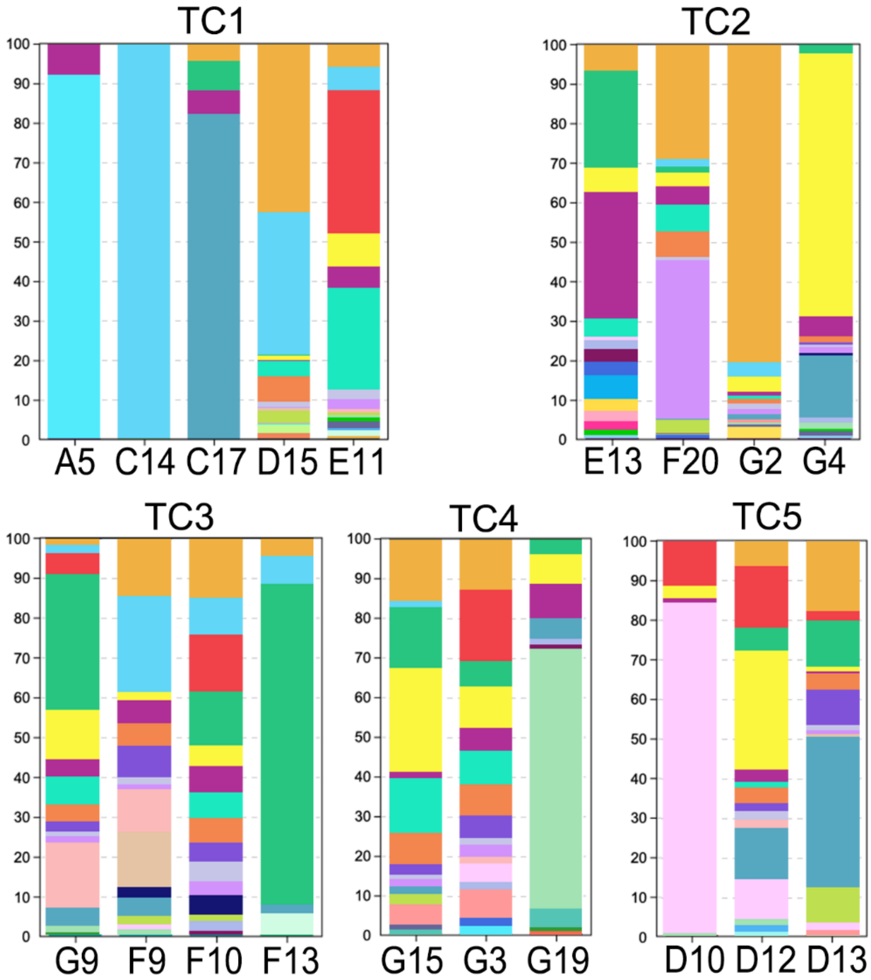

TC1
100
90
80
70
60
50
40
30
20
10
0
A5
C14
C17
D15
E11
TC2
E13
F20
G2
G4
TC3
G9
F9
F10
F13
TC4
G15
G3
G19
TC5
D10
D12
D13
